# Tumor-exosomes and leukocyte activation: an ambivalent crosstalk

**DOI:** 10.1186/1478-811X-10-37

**Published:** 2012-11-28

**Authors:** Daniela Zech, Sanyukta Rana, Markus W Büchler, Margot Zöller

**Affiliations:** 1 Department of Tumor Cell Biology, University Hospital of Surgery, Im Neuenheimer Feld 365, D-69120, Heidelberg, Germany; 2 University Hospital of Surgery, Heidelberg, Germany; 3 German Cancer Research Center, Heidelberg, Germany

**Keywords:** Tumor-exosomes, T cell activation, CTL, NK, Apoptosis, Leukocyte migration

## Abstract

**Background:**

Tumor-exosomes being reported to suppress or promote a cancer-directed immune response, we used exosomes of the rat pancreatic adenocarcinoma BSp73ASML (ASML) to evaluate, whether and which steps in immune response induction can be affected by tumor-exosomes and how the impaired responsiveness can be circumvented.

**Results:**

ASML-exosomes bind to and are taken up by all leukocyte subpopulations *in vivo* and *in vitro*, uptake by CD11b^+^ leukocytes exceeding that by T and B cells. ASML-exosomes affect leukocyte proliferation via reduced CD44v6 up-regulation and lck, ZAP70 and ERK1,2 phosphorylation, which can be compensated by dendritic cells (DC). ASML-exosomes do not support T_reg_. Yet, impaired activation of anti-apoptotic signals is accompanied by slightly increased apoptosis susceptibility. IgM secretion is unaffected; NK and CTL activity are strengthened, ASML-exosomes co-operating with DC in CTL activation. ASML-exosomes transiently interfere with leukocyte migration by occupying migration-promoting receptors CD44, CD49d, CD62L and CD54 during binding/internalization.

**Conclusion:**

ASML-exosomes might well serve as adjuvant in immunotherapy as they support leukocyte effector functions and have only a minor impact on leukocyte activation, which can be overridden by DC. However, exosome-induced modulation of immune cells relies, at least in part, on exosome uptake and message transfer. This implies that depending on the individual tumor's exosome composition, exosomes may distinctly affect the immune system. Nonetheless, whether immunotherapy can profit from using tumor-exosomes as adjuvant can easily be settled beforehand *in vitro*.

## Background

Exosomes, potent intercellular communicators that play a pivotal role in physiological and pathological processes [1] are found in all body fluids [2] and bind / are taken up by selected targets [3]. Exosomes contain function-competent proteins, mRNA and miRNA [1], which can severely affect the target cells [4,5]. These findings advocate for therapeutic use of exosomes, which is particularly appreciated in immunotherapy, as dendritic cell (DC)-exosomes, highly expressing MHCI, MHCII, CD80 and CD86, are fully equipped to initiate T cell activation [6,7].

DC-exosomes being a promising means for immunotherapy [6,8], hope has been dampened by tumor-exosomes interfering with immune response induction [9] such that tumor growth becomes promoted [10]. Tumor-exosomes can inhibit lymphocyte, predominantly CD4^+^ T cell proliferation in response to IL2, which is accompanied by impaired CD25 up-regulation and stronger suppressive activity of regulatory T cells (T_reg_), possibly due to exosome-associated TGFβ1 [11]. Impaired natural killer (NK) activity may rely on tumor-exosomes inhibiting activation of Stat5, Jak3, cyclinD3 expression and perforin release [12] or on blocking NK cells via NKG2D binding as far as exosomes express the relevant receptors. Exosomal MICA*008 also provokes a NKG2D-dependent reduction in NK cytotoxicity [13]. Tumor-exosomes affect T cells by inducing FAS-mediated apoptosis [14] and by enzymatic activity, which leads to extracellular adenosine production negatively modulating tumor infiltrating leukocytes (TIL) [15]. Tumor-exosomes may also suppress an antigen-specific response by inducing TGFβ1 and IL4 secretion and inhibiting DC maturation in draining lymph nodes [16]. Finally, tumor-exosomes can act as a decoy factor by capturing tumor-directed drugs like therapeutic antibodies as demonstrated for CD20 in hematological malignancies [17].

On the other hand, tumor-exosomes can support immune response induction. Exosomes express heat shock proteins (HSP) [18]. Stress-inducible HSP70 functions as an endogenous danger signal, promotes NK activation [19] and tumor cell lysis through granzyme B release. Exosomes recovered from heat-stressed tumor cells also were superior in inducing a tumor-antigen-specific cytotoxic T cell (CTL) response. Radiation-induced exosomal release of HSP72 increased IL6, TNFα, CTL and NK activity and induced costimulatory molecule expression in DC [20,21]. In line with this, vaccination with staphyloccocus enterotoxin A expressing tumor-exosomes significantly inhibited tumor growth and prolonged the survival time by increasing IL2 and IFNγ secretion and by promoting T helper (Th), CTL and NK activation [22]. Increased immunogenicity of exosomes from heat-stressed tumor cells is further strengthened by exosomal chemokines that attract and activate DC and T cells, whereby intratumor injection efficiently inhibited tumor growth [23]. Tumor-exosomes can be a strong immunogen such that tumor antigens, which are non-immunogenic when presented by tumor cells, induce a potent Th, CTL and B cell response and lead to a decrease in T_reg_, when presented by tumor-exosomes [24].

We described that exosomes of the metastasizing rat pancreatic adenocarcinoma ASML [25] support premetastatic niche preparation in draining lymph nodes [26]. Building on the proteome [26], mRNA and miRNA profile of ASML-exosomes http://www.ncbi.nlm.nih.gov/geo, accession No GSE34739], we elaborated changes in mRNA and protein expression in a lymph node stroma line that readily takes up ASML-exosomes. Besides others, ASML-exosomes induced up-regulation of several chemokines and adhesion molecules and down-regulation of molecules controlling / inhibiting cell cycle progression (S.Rana et al., submitted) that would rather favor than inhibit cell migration and proliferation. Based on these findings we asked, whether leukocytes, too, take-up ASML-exosomes and how they respond. ASML-exosomes do not inhibit immune response induction by DC, they hamper Th activation under suboptimal stimulatory conditions, but do not interfere with effector functions.

## Results

DC-derived exosomes are valuable therapeutics in cancer due to their capacity to induce T cell activation [6]. In contrast, tumor-exosomes may be immunosuppressive and counteract DC-exosomes [9]. To find modalities that would allow interfering with undue tumor-exosome activity, we explored tumor-exosome uptake and immune response modulation in a rat model.

### Leukocyte binding and uptake of tumor-exosomes

**Figure 1:**
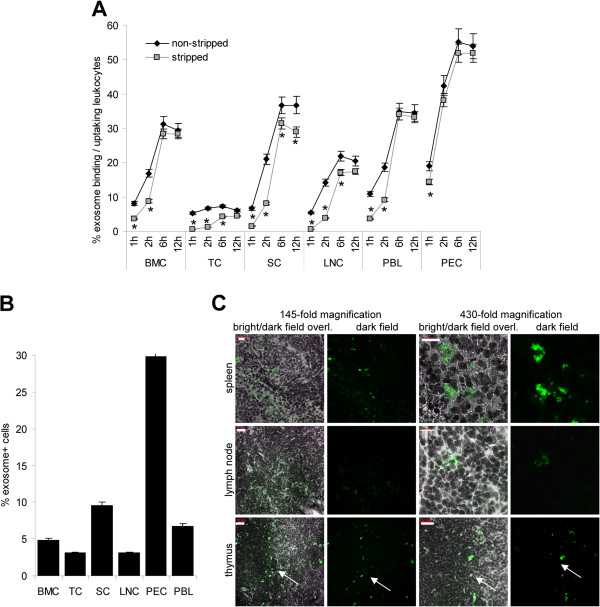
**Tumor-exosome uptake by leukocytes.** (**A**) BMC, TC, SC, LNC, PBL and PEC were incubated at 37°C with RhDHPE-labeled ASML-exosomes for the indicated periods. Exosome binding and uptake (fluorescence after 2 acid washes) was evaluated by flow-cytometry: Mean percent ± SD (3 experiments) of exosome^+^ leukocytes. Significant differences between exosome binding/uptake versus uptake: *. (**B**,**C**) SP-Dio_18_(3)-labeled exosomes (200 μg) were injected i.v. and hematopoietic organs were excised after 24 h: (**C**) Mean percent ± SD (3 rats) of exosome^+^ leukocytes evaluated by flow-cytometry. (**D**) Confocal microscopy showing representative examples of exosome^+^ cells in shock frozen lymph node, spleen and thymus (arrow: boundary cortex/medulla) sections (scale bar left: 20 μm, right: 10 μm). Tumor-exosomes bind and are taken up *in vitro* and *in vivo* by cells from all hematopoietic organs. Uptake is most rapid and abundant in PEC.

Binding and uptake of dye-labeled ASML-exosomes by leukocytes from central and peripheral lymphoid organs was evaluated by flow-cytometry. Exosome binding increased, when titrating exosomes from 10 μg/ml to 40 μg/ml and reached a plateau below 40 μg/ml (data not shown). To be in the saturating dose, 40 μg/ml of exosomes were used throughout. Exosomes readily bound within 1 h. However, leukocyte stripping by acid wash revealed that, with exception of peritoneal exudate cells (PEC), few leukocytes had taken-up exosomes after 1 h co-incubation. Exosome uptake increased until 6 h of co-incubation, when nearly all bound exosomes were taken-up. Highest recovery was observed in PEC, followed by spleen cells (SC), peripheral blood leukocytes (PBL), bone marrow cells (BMC) and lymph node cells (LNC). BMC behaved exceptionally, as a small population showed a very high level of exosome uptake, whereas thymocytes (TC) poorly took-up exosomes (Figure 1A, Additional file 1). ASML-exosomes were also taken-up by leukocytes *in vivo*. Dye-labeled exosome detection 24 h after i.v. injection confirmed preferential binding/uptake by PEC, followed by SC and PBL. It was low in lymph nodes and the thymus, exosomes in the thymus mostly being seen at the boundary between cortex and medulla (Figure 1B,C). Detection of dye-labeled exosomes after 48 h did not differ significantly from that after 24 h and started to decrease after 72 h (data not shown).

**Figure 2:**
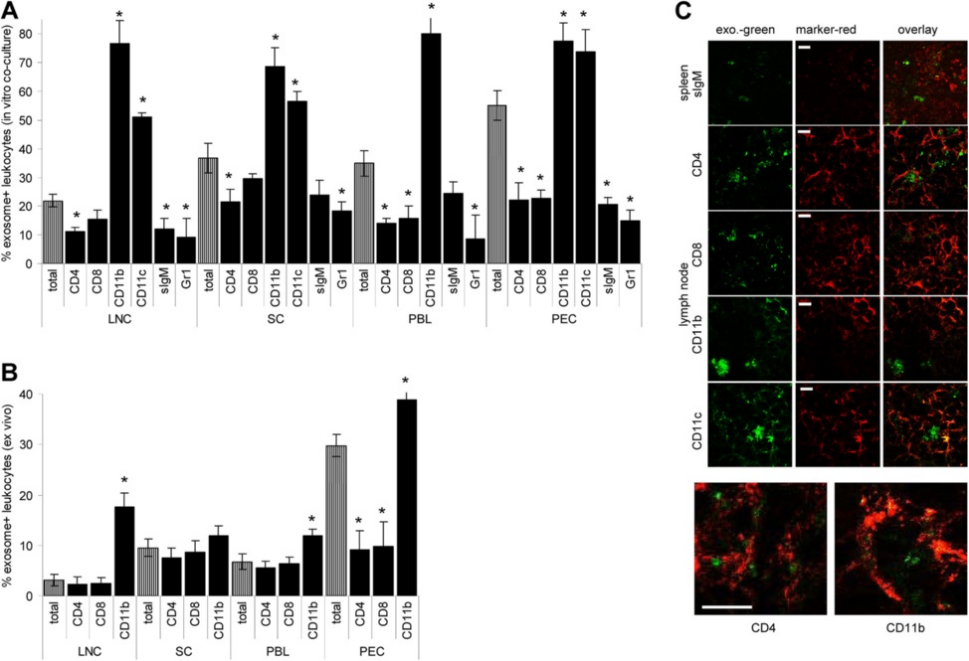
**Tumor-exosome uptake by leukocyte subpopulations.** (**A**) LNC, SC, PBL and PEC were incubated with RhDHPE-labeled ASML-exosomes for 6 h and stained with leukocyte subset-specific antibodies. The mean percent ± SD of marker^+^exosome^+^ / marker^+^ cells (3 experiments) is shown. (**B**,**C**) *In vivo* uptake of SP-Dio_18_(3) labeled ASML-exosomes 24 h after i.v. application: (**B**) flow-cytometry of dispersed cells. The mean percent ± SD of marker^+^exosome^+^ / marker^+^ cells (3 rats) is shown. (**C**) Confocal microscopy of shock frozen tissue sections (spleen sections were stained with anti-sIgM, lymph node sections with anti-CD4, -CD8, -CD11b and -CD11c. For lymph node sections stained with anti-CD4 and anti-CD11b a higher magnification is included (scale bar spleen: 20 μm, lymph nodes: 10 μm). (**A,B**) Significant differences in the % exosome^+^marker^+^ cells as compared to exosome^+^ cells in the total organ: *. Exosomes are taken up by cells of all major leukocyte subpopulations.

We next explored *in vitro* and *in vivo*, which subpopulations in peripheral lymphoid organs preferentially take-up ASML-exosomes. *In vitro* uptake was evaluated after 4 h co-incubation. With the exception of macrophages (Mϕ) (CD11b^+^) and DC (CD11c^+^), which most efficiently took-up tumor-exosomes, uptake by CD4^+^, CD8^+^ and sIgM^+^ lymphocytes was in a comparable range and uptake by granulocytes was lower (Figure 2A). A similar profile of exosome uptake was seen 24 h after *in vivo* application. Counterstaining of spleen and lymph node sections revealed that exosomes co-localized only with CD11b and CD11c, though exosomes were also seen in CD4^+^ and CD8^+^ cells (Figure 2B,C). This suggested that CD11b and CD11c, but not CD4 or CD8 are involved in exosome uptake.

**Figure 3:**
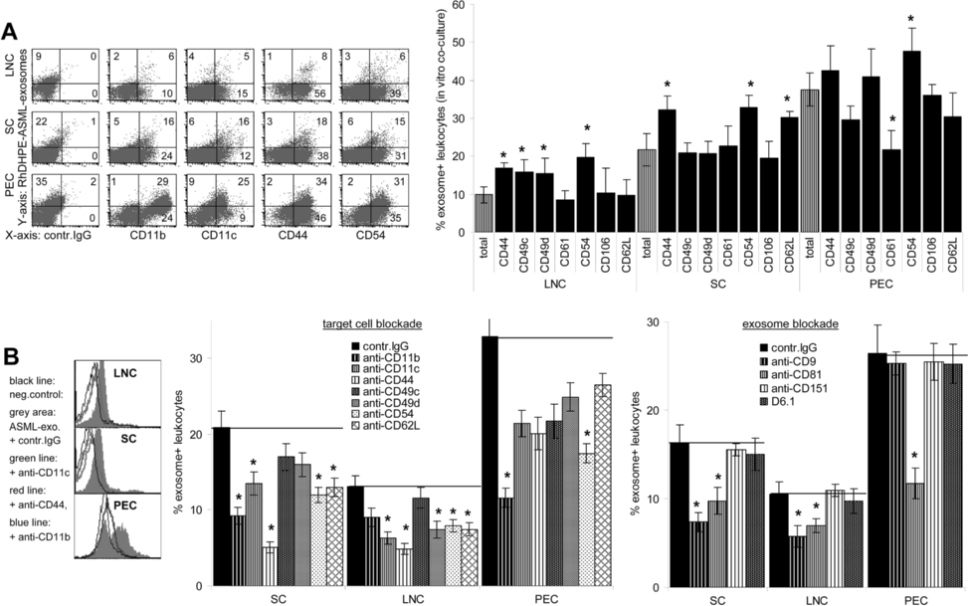
**Adhesion molecules engaged in tumor-exosome uptake.** (**A**) Cells as in (Figure 2A) were stained with adhesion molecule-specific antibodies: representative examples and mean percent ± SD of marker^+^exosome^+^ / marker^+^ cells (3 experiments). (**B**) LNC, SC and PEC or exosomes were pre-incubated with the indicated antibodies (30 min, 4°C). After washing, cells were co-incubated with dye-labeled exosomes 2h, 4°C: representative examples and mean percent ± SD (3 experiments) of exosome^+^ cells. (**A**) Significant differences in the % exosome^+^marker^+^ cells as compared to exosome^+^ cells in the total organ: *, (**B**) significant differences compared to control IgG treatment: *. There is evidence for an engagement of CD11b, CD11c, CD44, CD49d, CD54 and CD62L in exosome uptake.

Previous work showing exosomal tetraspanin-integrin complexes to bind to integrin receptors on stroma and endothelial cells [27,28], we asked whether ASML-exosomes also bind to leukocyte adhesion molecules. Besides CD11b^+^ and CD11c^+^ leukocytes, exosomes were preferentially incorporated into CD11a^+^, CD44^+^, CD49d^+^ and CD54^+^ leukocytes. CD62L^+^ SC also showed a relative increase in tumor-exosome uptake (Figure 3A). To investigate whether these adhesion molecules are directly engaged, leukocytes were pre-incubated with antibody. To avoid uptake, antibody blocking studies were performed at 4°C (30 min). A blockade of CD11b, CD11c, CD44, CD49d, CD54 and CD62L on LNC and SC interfered with exosome binding (2 h, 4°C). Binding to PEC was most strongly inhibited by anti-CD11b and anti-CD54. At the level of the exosomes, a blockade of the tetraspanins CD9 and CD81 interfered with binding (Figure 3B).

Taken together, (i) tumor-exosomes bind and are taken-up *in vitro* and *in vivo* by T cells, NK, B cells, DC, Mϕ and granulocytes; (ii) leukocyte subpopulations differ in tumor-exosome uptake, which for ASML-exosomes is highest for PEC and lowest for granulocytes; (iii) differences in tumor-exosome uptake depend on the availability of leukocyte ligands for exosomal receptors, where CD11b, CD11c, CD44, CD49d, CD54 and CD62L are engaged in ASML-exosome binding; (iv) as previously shown [28], exosomes bind via tetraspanin complexes.

### Tumor-exosomes can inhibit leukocyte proliferation and weaken apoptosis resistance

Exosome binding can initiate signal transduction via activation of target cell ligands. However, exosomes also are taken-up by target cells and the uptaken exosomes exert long-lasting effects on their targets [29]. Furthermore, as exosome binding and uptake proceed concomitantly, it is difficult to define effects initiated exclusively by binding. Finally, the impact of ASML-exosomes on a lymph node stroma line was analyzed in detail showing that proteins, mRNA and miRNA are transferred, target cells being mostly affected by exosomal miRNA [26], http://www.ncbi.nlm.nih.gov/geo, accession No GSE34739, Rana et al., submitted. For these reasons we evaluated the impact of uptaken exosomes on leukocyte activity, the exosomes being present throughout the culture period, but at least for 6 h.

**Figure 4:**
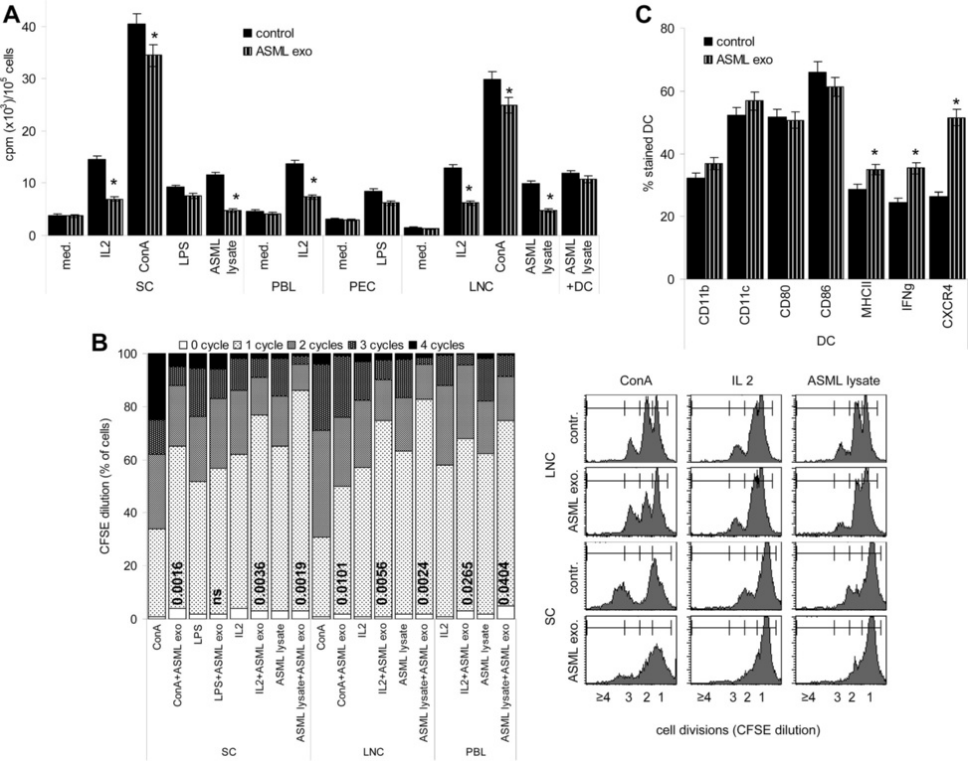
**ASML-exosomes and leukocyte proliferation.** Lymphocytes were stimulated for 72 h as indicated with/without ASML-exosomes. Where indicated, cultures additionally contained ASML lysate-pulsed DC (LNC:DC = 10:1). (**A**) Mean ± SD (triplicates) of ^3^H-thymidine incorporation. (**B**) Examples of CFSE dilution in LNC and SC cultured for 72 h and mean percentage (triplicates) of cells that did progress through 0–4 cycles. Significant differences to cultures not containing ASML-exosomes are shown. (**C**) BMC-derived DC were cultured as described in MM. During the last 24 h of culture in the presence of LPS, ASML-exosomes were added where indicated: Mean percent ± SD (3 experiments) of CD11b^+^, CD11c^+^, CD80^+^, CD86^+^, MHCII^+^, IFNγ^+^ and CXCR4^+^ cells (flow-cytometry). (**A,C**) Significant differences in the presence of ASML-exosomes: *. Exosomes inhibit lymphocyte proliferation, which can be circumvented by activated DC.

Though ASML-exosomes did not promote a major redistribution of T cell subsets (Additional file 2), proliferative activity, evaluated by ^3^H-thymidine incorporation, was impaired. The response to IL2 and tumor-lysate (as nominal antigen) was more strongly affected than the response to the polyclonal T cell stimulus ConA. Low proliferative activity in the absence of a stimulus and in response to LPS was not affected. CFSE dilution confirmed these findings. Notably, when LNC were supported by antigen-loaded DC, proliferation-suppressive activity of ASML-exosomes was effaced (Figure 4A,B). Further, tumor-exosomes did not affect DC maturation. CD11c, CD80 and CD86 expression was unimpaired and MHCII, IFNγ and CXCR4 expression was augmented, when DC were matured in the presence of ASML-exosomes (Figure 4C).

Reduced proliferative activity could have been due to myeloid-derived suppressor cell (MDSC) or T_reg_ expansion, apoptosis induction or impaired T cell activation by ASML-exosomes.

**Figure 5:**
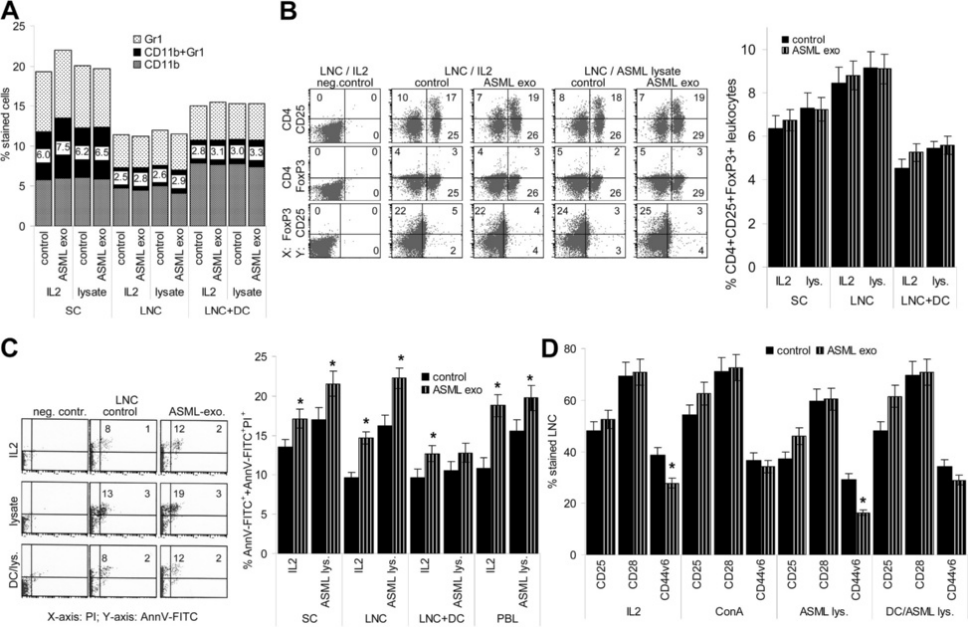
**ASML-exosomes, immunosuppression, apoptosis and activation markers.** Leukocytes were stimulated as described in Figure 4. (**A**) Mean percent (3 experiments) of Gr1^+^, CD11b^+^ and Gr1^+^CD11b^+^ (MDSC) cells. (**B**) examples of CD4^+^CD25^+^, CD4^+^FoxP3^+^ and CD25^+^FoxP3^+^ cells and mean percent ± SD (3 experiments) of CD4^+^CD25^+^FoxP3^+^ cells. (**C**) Representative examples of AnnexinV/PI staining and mean percent ± SD (3 experiments) of AnnV-FITC^+^/AnnV-FITC^+^/PI^+^ cells. (**D**) Mean percent ± SD (3 experiments) of CD25^+^, CD28^+^ and CD44v6^+^ cells. (**C,D**) Significant differences in the presence of ASML-exosomes: *. There is no evidence for ASML-exosomes affecting MDSC or T_reg_. However, apoptosis susceptibility is slightly increased and expansion of CD44v6^+^ cells is impaired.

Independent of the presence of DC, tumor-exosomes did not promote MDSC or T_reg_ expansion (Figure 5A,B). However, ASML-exosome-treated lymphocytes showed slightly increased apoptosis susceptibility (Figure 5C). Furthermore, up-regulation of the accessory molecule CD44v6 [30], though not of CD25 and CD28, was reduced in IL2 or ASML-lysate stimulated cells. CD44v6 expression was not significantly reduced in the presence of ConA or DC (Figure 5D).

**Figure 6:**
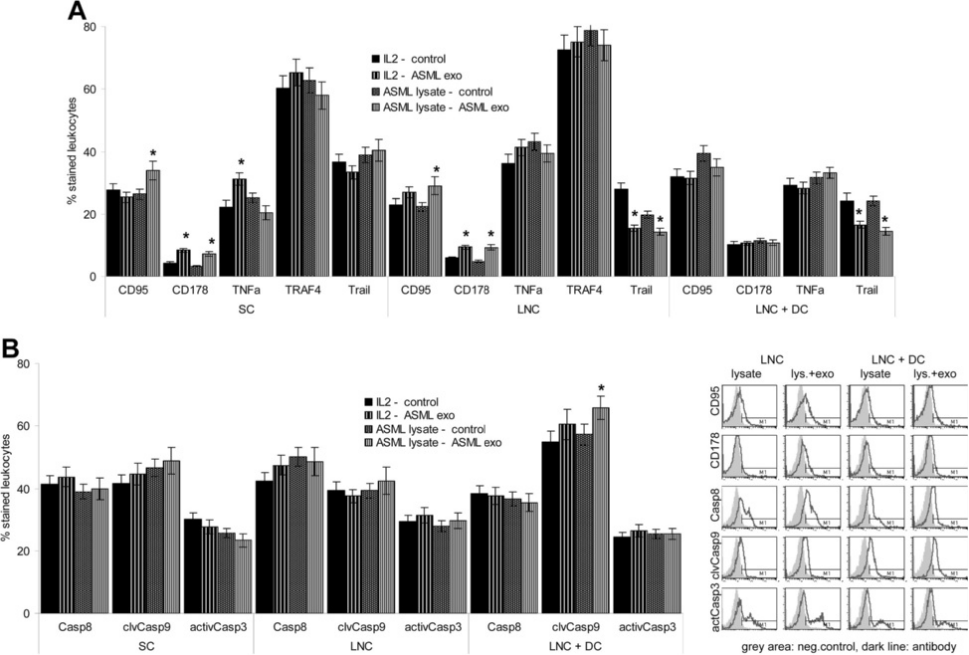
**ASML-exosomes and death receptors.** Leukocytes were stimulated as described in Figure 4. Expression of (**A**) death receptors and (**B**) caspases was evaluated by flow-cytometry. Representative examples and mean percent ± SD (3 experiments) of stained cells; significant differences in the presence of ASML-exosomes: *. ASML-exosomes do not promote caspase activation.

**Figure 7:**
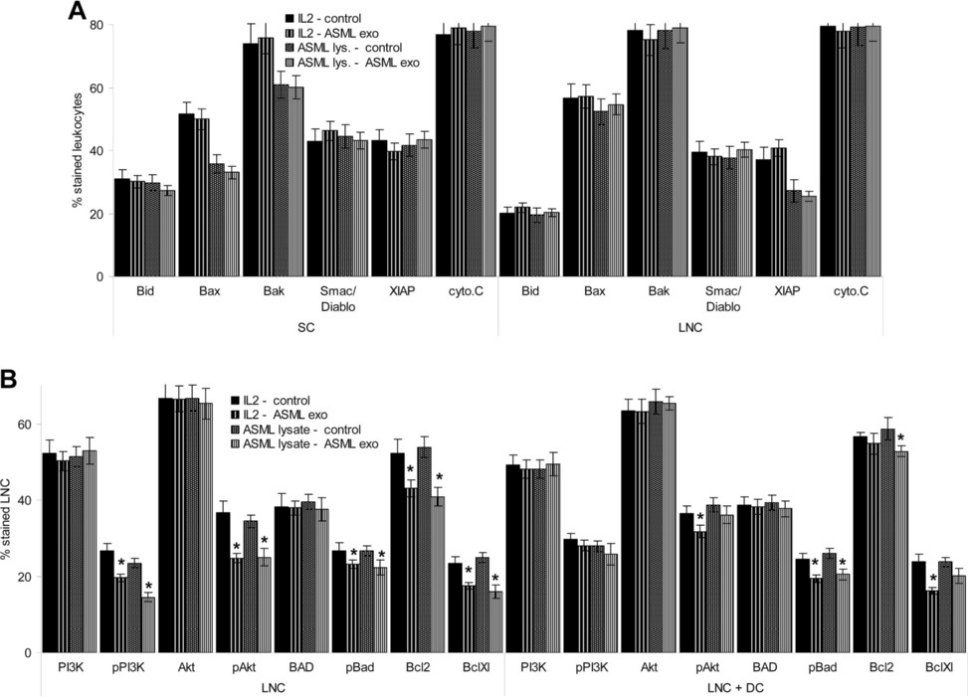
**ASML-exosomes and activation of pro- and anti-apoptotic molecules.** Leukocytes were stimulated as described in Figure 4. Expression of (**A**) pro-apoptotic molecules and (**B**) anti-apoptotic molecules was evaluated by flow-cytometry. Mean percent ± SD (3 experiments) of stained cells; significant differences in the presence of ASML-exosomes: *. ASML-exosomes slightly affect activation of the PI3K/Akt pathway.

Having excluded MDSC and T_reg_ to account for reduced proliferative activity in the presence of ASML-exosomes, we searched for the mechanism underlying the slightly increased apoptosis susceptibility. CD95L (CD178) expression was slightly increased in LNC and SC co-cultured with ASML-exosomes that was not seen in the presence of DC. TNFα expression was only increased in SC, TRAF4 expression was not affected and Trail expression was reduced (Figure 6A). Despite slight CD95L up-regulation, Caspase8 expression, Caspase9 cleavage and Caspase3 activation were unaltered (Figure 6B), which excludes CD95L up-regulation to contribute to lymphocyte suicide. Searching for changes in the mitochondrial pathway of apoptosis and apoptosis protection revealed unaffected Bax, Bid, Bak, Smac/Diablo, XIAP and cytochromeC expression in ASML-exosome-treated LNC and SC (Figure 7A). Instead, PI3K and Akt phosphorylation was slightly, but significantly reduced. BAD phosphorylation and Bcl2 and BclXl expression also was slightly affected in LNC and SC and, though mitigated, in cultures containing DC (Figure 7B, Additional file 3A). Thus, impaired anti-apoptotic signaling may account for the slightly increased lymphocyte apoptosis-susceptibility.

**Figure 8:**
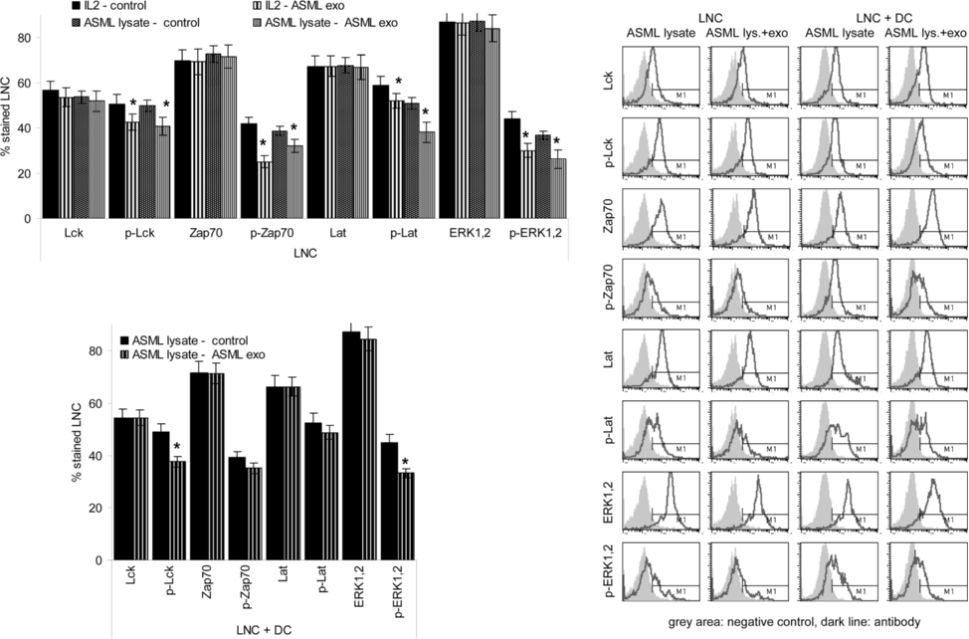
**ASML-exosomes and TCR activation.** Leukocytes were stimulated as described in Figure 4. Expression of molecules involved in TCR signaling was evaluated by flow-cytometry. Representative examples and mean percent ± SD (3 experiments) of stained cells; significant differences in the presence of ASML-exosomes: *. In the presence of ASML-exosomes activation of lck and TCR downstream kinases of the MAPK pathway is impaired. Both effects are strongly mitigated in the presence of DC.

Reduced CD44v6 expression in LNC and SC was accompanied by a reduction in Lck, ZAP70 and Lat phosphorylation and impaired activation of the MAPK (ERK1,2) cascade. The JNK pathway (JNK, c-jun) and NFκB (IκB phosphorylation) were not affected. When activation of exosome-treated lymphocytes was supported by ASML-lysate-pulsed DC, Lck and ERK1,2 phosphorylation was still reduced, but ZAP70 and LAT phosphorylation was unimpaired (Figure 8, Additional file 3B).

Taken together, tumor-exosomes affected lymphocyte proliferation, most pronounced in response to IL2. Reduced proliferation was not due to MDSC or T_reg_ expansion. Instead, reduced CD44v6 expression could account for impaired activation of the PI3K/Akt pathway [31,32] and, as CD44 associates with Lck [33], for mitigated T cell activation, which had consequences mainly on MAPK pathway activation. However, CD28 expression and DC activation not being affected, reduced CD44v6 expression was mostly compensated in the presence of DC.

### Tumor-exosomes support effector lymphocytes

**Figure 9:**
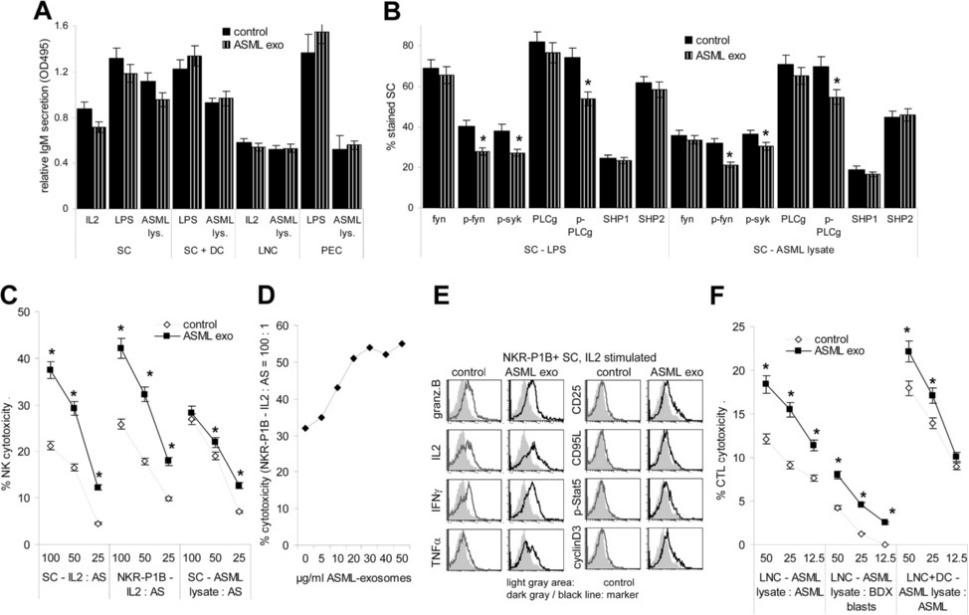
**ASML-exosomes and effector lymphocytes.** (**A**,**B**) SC, LNC and PEC were stimulated with IL2, LPS or ASML lysate with/without ASML-exosomes: (**A**) Supernatants were harvested after 4d to evaluate IgM secretion by ELISA. (**B**) Expression of B cell activation-related signal transduction molecules was evaluated by flow-cytometry after 2d (mean values ± SD, 3 experiments). (**C**) SC and NKR-P1B^+^ cells were cultured in the presence of 100U IL2/ml or ASML-lysate for 2d. NK cytotoxicity was evaluated with ^3^H-thymidine labeled AS target cells. (**D**) NKR-P1B^+^ cells were cultured in the presence of 100U IL2/ml and titrated amounts of ASML-exosomes. Cytotoxicity was evaluated as in (**C**) after 2d of co-culture. (**E**) NKR-P1B^+^ cells were cultured in the presence of 100U IL2/ml and 40 μg/ml ASML-exosomes for 2d. Expression of granzymeB, IL2, IFNγ, TNFα, CD25, CD95L, p-Stat5 and CyclinD3 was evaluated by flow cytometry. (**F**) LNC were cultured in the presence of ASML-lysate for 8d. Where indicated, cultures contained ASML lysate-loaded DC. CTL activity was evaluated with ^3^H-thymidine labeled ASML and BDX blast target cells: (**C,D,F**) Mean percent ± SD (triplicates) of cytotoxicity at the indicated E:T ratios. (**A-F**) Significant differences in cultures containing ASML-exosomes: *. Tumor-exosomes do not hamper a primary B cells response to T cell-dependent or T cell-independent stimuli. But, activation of fyn, syk and PLCγ are slightly impaired. Tumor-exosomes strengthen NK and CTL activity.

ASML-exosomes did not affect B cell proliferation in response to LPS (Figure 4A) and IgM secretion was not reduced in IL2, LPS and ASML-lysate stimulated SC, LNC, PEC or in DC-supported SC co-cultured with ASML-exosomes (Figure 9A). Neither sIgM nor CD81 expression was affected (Additional file 4). Nonetheless, fyn, syk and PLCγ phosphorylation was slightly reduced in SC, but not in PEC co-cultured with ASML-exosomes. There was no evidence for concomitant SHP up-regulation (Figure 9B).

Tumor-exosomes promoted NK and CTL activity. NK activity, evaluated against highly NK-susceptible AS cells [34] was most strongly supported by ASML-exosomes in response to IL2. When co-cultured with NK-enriched NKR-P1B^+^ SC, even 10 μg/ml ASML-exosomes sufficed for an increase in cytotoxic activity. Furthermore, granzymeB, IL2, IFNγ, TNFα, CD25 and weakly CD95L expression was upregulated in IL-2 stimulated NKR-P1B + SC, when cultured in the presence of ASML exosomes. Stat5 phosphorylation and cyclinD3 expression was also slightly increased (Figure 9C-E). ASML-exosomes also strengthened CTL activity against NK-resistant ASML cells, and exerted co-operative activity with tumor-lysate-loaded DC, which by themselves stimulated CTL activation. ASML-exosomes also promoted, albeit weakly, activation of auto-reactive CTL (syngeneic blasts) (Figure 9F).

Though primary B cell responses were unimpaired, the discrete differences in activation of signaling molecules associated with B cell activation suggest ASML-exosomes to possibly affect B cell response regulation. ASML-exosomes strongly promoted NK and CTL activity, the latter in co-operation with DC.

### Tumor-exosomes and T cell migration

A tumor-specific immune response can only become effective, when activated lymphocytes reach the tumor [35]. Thus, an impact of ASML-exosomes on T cell migration could be a hindrance.

**Figure 10:**
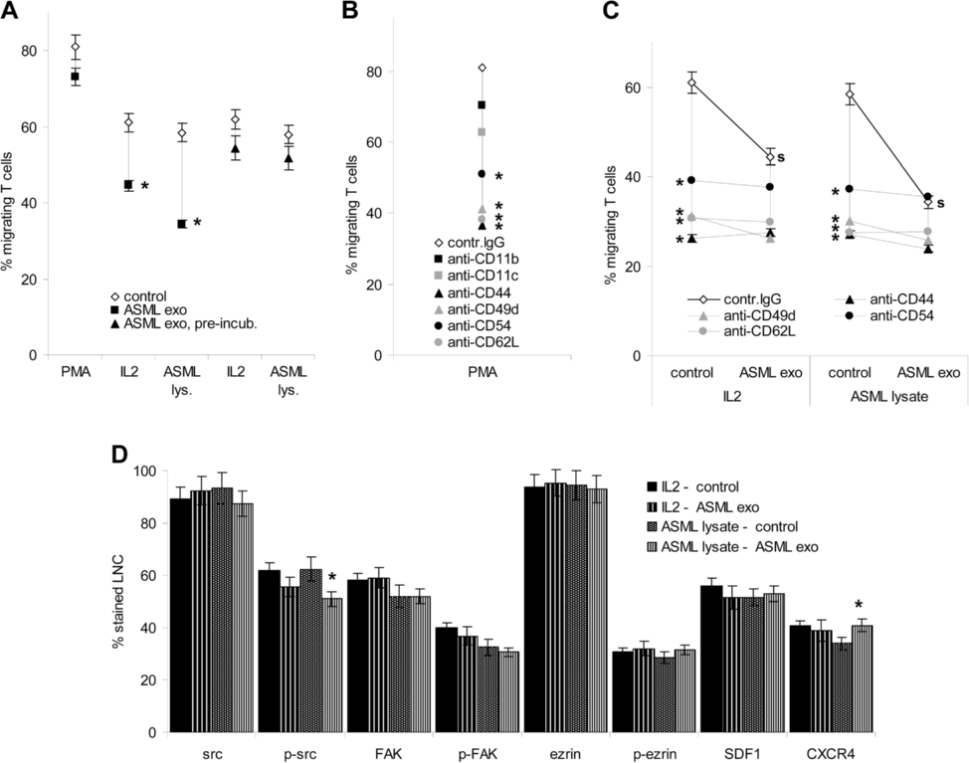
**ASML-exosomes and T cell migration.** T cells were stimulated as indicated for 24 h. (**A**-**C**) Stimulated T cells were seeded in RPMI/1%FCS in the upper part of a Boyden chamber, the lower part contained RPMI/20%FCS. Where indicated, T cells stimulated in the presence of ASML-exosomes were washed, evaluating migration in the absence of ASML-exosomes. The percentage of migrating cells was evaluated after 4 h at 37°C. (**B**) T cells were stimulated for 24 h with PMA. Before migration, cells were incubated with the indicated antibodies (30 min, 4°C). (**C**) T cells were stimulated, washed and incubated with the indicated antibodies (30 min, 4°C). Migration was evaluated in the presence/absence of ASML-exosomes. (**A-C**) Mean percent ± SD (triplicates, 3 experiments) of migrating cells. (**A**) Significant differences in cultures containing or pre-incubated with ASML-exosomes: *, (**B,C**) significant antibody inhibition: *, (**C**) significant inhibition in the presence of ASML-exosomes: **s**. (**D**) Migration-related signaling molecule including SDF1 and CXCR4 expression was evaluated by flow-cytometry. Mean percent ± SD (3 experiments) of stained cells, significant differences in cultures containing ASML-exosomes: *. Tumor-exosomes affect T cell migration. This is due to a blockade of migration-relevant adhesion molecules engaged in exosome uptake. Up-taken exosomes hardly affect T cell migration.

When evaluating magnetic bead-enriched lymph node T cell migration in the presence of ASML-exosomes, PMA-stimulated T cell migration was hardly affected, but that of IL2 and ASML-lysate stimulated T cells was mitigated (Figure 10A). As adhesion molecule expression was not or minimally (CD18 and CD62L) affected (Additional file 5), we questioned whether ASML-exosomes may transiently occupy adhesion molecules required for migration. Indeed, migration of lymphocytes that had been pretreated, but did not contain ASML-exosomes during migration was far less reduced (Figure 10A). This finding pointing towards impaired availability of adhesion molecules contributing to the ASML-exosome-mediated blockade in T cell migration, we controlled, which antibodies interfere with migration in the absence of exosomes. T cell migration was strongly affected by anti-CD49d, anti-CD44, anti-CD62L and anti-CD54 (Figure 10B). As CD44, CD49d, CD62L and CD54 are engaged in exosome binding (Figure 3B), we speculated that exosomes should not be inhibitory for T cells pre-incubated with these antibodies. In fact, ASML-exosomes did not further reduce migration of antibody-pre-incubated T cells (Figure 10C). From there we conclude that the inhibitory effect of ASML-exosomes is mostly due to transient occupancy of adhesion molecules required for migration. In line with this, SDF1 and CXCR4 expression was not reduced, FAK and ezrin phosphorylation was not and src phosphorylation was only slightly impaired in ASML-exosome treated T cells (Figure 10D).

Thus, exosome binding and/or the internalization of the exosome ligand(s) during uptake mostly accounts for transiently impaired T cell migration.

### Tumor-exosomes and leukocyte activation *in vivo*

To control for the impact of tumor-exosomes on leukocyte activation *in vivo*, BDX rats received subcutaneous injections of 2×10^6^ ASML lysate-loaded DC and 2-times/week 500 μg ASML-exosomes (i.v.). Rats were bled and sacrificed 3d after the 3^rd^ DC injection, the draining axillary and inguinal lymph nodes and the spleen were excised and analyzed.

**Figure 11:**
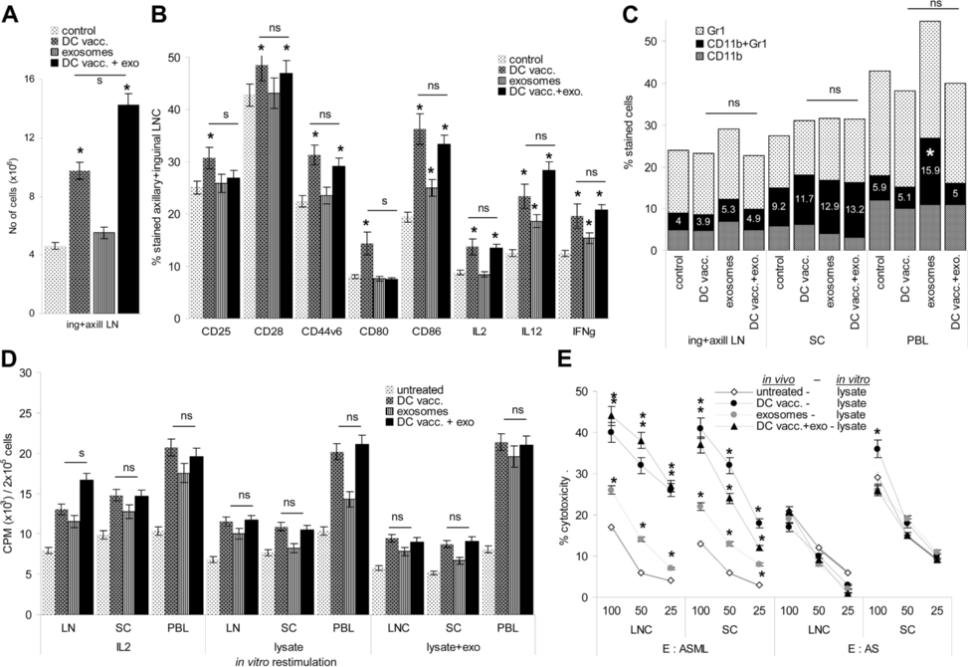
***In vivo *impact of ASML-exosomes on leukocyte activation.** Rats received 3-times 2x10^6^ DC, subcutaneously and/or 7-times 500 μg ASML-exosomes, i.v. as described in MM. Rats were sacrificed 3d after the 3^rd^ DC application to analyze the draining LNC, SC and PBL. (**A**) Number of draining LNC (mean ± SD, 3 rats), (**B**) leukocyte activation markers, IL2, IL12 and IFNγ expression (flow cytometry, mean ± SD, 3 rats and representative examples), (**C**) MDSC (flow cytometry, mean ± SD, 3 rats), (**D**) ^3^H-thymidine incorporation after 3d *in vitro* culture and (**E**) cytotoxic activity against ASML and AS (NK susceptible) targets after 10d *in vitro* culture in the presence of ASML lysate (mean ± SD, triplicates). (**A-E**) Significant differences to lymphocytes from untreated rats: *, differences between lymphocytes from rats receiving DC or DC plus ASML-exosomes are indicated as ns (not significant) or s (significant, p <0.01). *In vivo*, ASML-exosomes support recruitment and/or proliferation of draining LNC and CD11b, CD86, IL12 and IFNγ expression. Despite an increase in MDSC in the peripheral blood, ASML-exosomes support DC vaccination-induced T cell expansion and cytotoxic activity.

DC vaccination was accompanied by an >2-fold increase in the number of draining LNC, which was further increased, when rats received concomitantly ASML-exosomes (Figure 11A). DC vaccination promoted a slight expansion of CD4^+^, CD8^+^ and CD11c^+^ cells. Exosomes induced expansion of CD11b^+^ cells. DC vaccination also induced CD25, CD28, CD44v6, CD80, CD86, IL2, IL12 and IFNγ upregulation. Only CD25 and CD80 upregulation was not seen in rats concomitantly receiving ASML-exosomes. (Figure 11B, Additional file 6A). Exosome application did not affect T_reg_ and an expansion of MDSC was only seen in the peripheral blood, though not in rats concomitantly receiving DC (Figure 11C, Additional file 6B). Unexpectedly, ASML-exosomes did not inhibit, but rather promoted LNC, SC and, most pronounced, PBL proliferation *in vivo*. High proliferative activity of lymphocytes from rats receiving DC was not affected by concomitant ASML-exosome application. This was independent of whether lymphocytes were restimulated *in vitro* by IL2, ASML lysate or ASML lysate plus exosomes (Figure 11D). The cytotoxic activity of LNC and SC also was unimpaired in rats receiving lysate-pulsed DC plus ASML-exosomes and lymphocytes from rats treated with ASML-exosomes showed increased CTL activity compared to untreated controls. NK activity was hardly affected by DC, exosome or DC plus exosome treatment (Figure 11E).

With the exception of an increase in MDSC in the peripheral blood of ASML-exosome-treated rats, these *ex vivo* data confirm the results obtained after *in vitro* co-culture and show that *in vivo* ASML-exosomes support immune response induction by tumor-lysate-loaded DC.

## Discussion

Tumor-exosomes are discussed to possibly provide a hindrance in immunotherapy by suppressing immune response induction as well as immune effector cells [9]. However, opposing findings have also been observed [23,36]. We approached the question using exosomes of a highly metastatic rat pancreatic adenocarcinoma, where pancreatic adenocarcinoma are known for their intense interaction with the surrounding tissue including TIL [37]. Though ASML-exosomes interfere with leukocyte activation *in vitro*, they did not affect or supported effector cells and a reduction in T cell migration was transient. These findings - not excluding immunosuppression by other tumor-derived vesicles – argue against tumor-exosomes being a hindrance in immunotherapy.

### Tumor exosome binding and uptake

Tumor-exosomes bind and are taken-up by leukocytes in central and peripheral lymphoid organs, though binding/uptake varies considerably being highest for PEC and lowest for TC. As a very similar profile of binding/uptake was observed *in vitro* and *in vivo*, the low uptake by TC cannot exclusively rely on poor accessibility of the thymus. Also, the high recovery of exosomes in PEC cannot be explained by "first station" capture that would be the spleen after i.v. injection. Thus, exosome uptake is a directed process that is particularly efficient in Mϕ and DC.

Exosome uptake by solid organ-derived cells mostly proceeds via binding of tetraspanin-associated adhesion molecules to their target cell ligands [27,28]. Antibody-blocking studies revealed CD11 integrins, CD49d, CD44, CD54 and CD62L to be engaged in exosome uptake, where up-regulated CD44 and CD49d expression on activated lymphocytes [38] and CD11c on activated DC [39] could facilitate exosome binding. Similar to AS-exosomes (28), ASML-exosomes mostly use (CD9 and CD81) tetraspanin complexes for leukocyte binding. Uptake by Mϕ may proceed distinctly from that by lymphocytes. Binding of Annexins to scavenger receptors can play a role in exosome uptake [40,41]. The more rapid exosome uptake by Mϕ that is mostly inhibited by anti-CD11b argues for Annexin possibly being important in exosome uptake by Mϕ.

Taken together, tumor-exosomes uptake by CD11b^+^ Mϕ may preferentially proceed via scavenger receptors, uptake by lymphocytes and dendritic cells via adhesion molecule ligands for exosomal receptors in tetraspanin complexes. Exosome uptake by leukocytes will vary according to the leukocyte ligands as well as exosomal (tetraspanin)-adhesion molecule complexes.

### Tumor-exosomes and lymphocyte activation

Proliferation of T cells stimulated in the presence of tumor-exosomes was reduced, most pronounced when stimulated by IL2.

Tumor-exosomes can strengthen MDSC [42,43] and T_reg_ expansion/activation [11,44,45], but also impair T_reg_ expansion [24]. We did not observe any effect on T_reg_ and only *in vivo* an increase in MDSC in the peripheral blood. These findings exclude ASML-exosomes to impair lymphocyte activation due to active suppression.

Tumor-exosomes can induce lymphocyte apoptosis via CD95L and TRAIL [46,47]. Although there was a slight increase in CD95L^+^ lymphocytes and in early (AnnV^+^/PI^-^) apoptotic lymphocytes, co-culture with ASML-exosomes did not suffice to initiate receptor-mediated apoptosis or to activate the mitochondrial apoptosis pathway. Instead, ASML-exosomes slightly affected activation of anti-apoptotic molecules of the PI3K/Akt pathway. This could well be a consequence of impaired CD44v6 upregulation, CD44v6 supporting liberation of Bcl2 and Bcl-Xl from BAD via activation of the MAPK pathway (31,32).

Proliferation being most strongly suppressed in response to IL2, but not in the presence of DC, also points towards an impact of ASML-exosomes on accessory molecule, particularly CD44 activation. During lymphocyte activation CD44 associates with lck, which becomes phosphorylated and contributes to ZAP70 phosphorylation [48]. CD44v6 also can directly promote MAPK pathway activation [26,49]. In cultures containing ASML-exosomes, lck, ZAP70, LAT and ERK1,2 phosphorylation was significantly, though not strongly reduced, possibly due to support by other accessory molecules like CD25, CD28 or CD40L. Notably, in co-cultures with DC, lck and ERK1,2 phosphorylation, but not ZAP70 and LAT phosphorylation remained reduced. Thus, in the presence of antigen-loaded DC showing high CD40 expression, impaired CD44v6-initiated signaling becomes invalidated.

Taken together, tumor-exosomes inhibit Th expansion/activation in response to IL2. The inhibitory effect can be circumvented by provision of appropriate second signals, e.g. via DC. There was no evidence for exosomes affecting signals directly initiated by TCR engagement. In line with this, no changes in signal transduction molecule activation were seen after 1 h co-incubation of leukocytes with exosomes (data not shown). Moreover, ASML-exosomes hardly promote apoptosis and not immunosuppression, which findings were confirmed *in vivo*.

### Tumor-exosomes and lymphocyte effector functions

ASML-exosomes did not interfere with a primary B cell response. Nonetheless, fyn, syk and PLCγ phosphorylation was slightly reduced. Although ASML-exosomes did not induce activation of phosphatases, which can dephosphorylate syk and thereby account for PLCγ down-regulation [50], we cannot exclude that ASML-exosomes may have some bearing on B cell response regulation.

ASML-exosomes strongly stimulated NK activity in the presence of IL2. Tumor-exosomes can suppress NK activity by inhibition of JAK/STAT pathway signal transduction, reduced perforin release or a blockade of NK activating receptors [12,13], but also can stimulate NK cells by high HSP expression [19,21], as known for ASML-exosomes [26]. Irrespective of the constitutively high NK activity of BDX rats [51], pronounced induction of GranzymeB and IFNγ expression in NKR-P1B cells in the presence of ASML-exosomes argues for exosomes-supported NK activation rather than suppression. The same accounts for CTL, where ASML-exosomes efficiently stimulated tumor-specific and only to a minor degree, autoreactive CTL. Importantly, ASML-exosomes strengthened DC-supported CTL activation, probably due to tumor-exosome uptake by DC [36].

It should be mentioned that according to our protocol of prolonged leukocyte – exosome co-incubation, we missed binding-initiated activation of signaling cascades and changes in signaling pathway activation could likely be due to transferred exosomal proteins, mRNA or miRNA [5,7], where exosomal miRNA might be dominating (Rana et al., submitted). However, irrespective of whether exosomes binding or uptake slightly mitigated activation of several signal transduction pathways, ASML-exosomes did not hamper or stimulated lymphocyte effector functions *in vitro* and lymphocyte activation was not impaired *in vivo*. Thus, increased NK and CTL activity argues for ASML-exosomes as an immunotherapy supporting regimen.

### Tumor-exosomes and leukocyte migration

As activated leukocytes need to reach the tumor, the impact of ASML-exosomes on T cell migration became important. Migration was significantly impaired only when leukocytes were exposed to exosome during migration and antibody blocking studies confirmed that exosomes only transiently interfered with lymphocyte migration by occupying or co-internalizing with their migration-relevant ligands, CD44, CD49d, CD62L and CD54 [38,52]. Instead, uptaken exosomes did not affect leukocyte migration. These findings are in line with ASML-exosomes not affecting src, FAK and ezrin phosphorylation and SDF1 and CXCR4 expression. Upregulated CXCR4 expression in draining LNC after i.v. application of ASML-exosomes and leukocyte recruitment into the draining node after subcutaneous ASML-exosome application [26] also argue against exosomes hampering leukocyte migration *in vivo*.

## Conclusion

ASML-exosomes were taken up by leukocytes and interfered, though not severely, with T cell expansion *in vitro*, which could be circumvented by supportive regimens like DC. They did not promote T_reg_ expansion and only *in vivo* a slight increase in MDSC was seen. T cell migration was only transiently impaired during exosome uptake. Importantly, ASML-exosomes supported effector cells and cooperated with DC [23], whereby tumor-exosomes can become a stronger immunogen than a membrane-bound or soluble tumor antigen [24,36]. In addition, tumor-exosomes can provide a reliable source of tumor antigens in tumors where immunogenic entities are unknown. Nonetheless, as exosome-mediated intercellular communication may be dominated by transferred miRNA [5], exosomes of different tumors could distinctly affect the immune system, which can be easily evaluated *in vitro* before vaccinating with tumor-exosomes.

## Methods

### Cell lines

The rat pancreatic adenocarcinoma lines ASML and BSp73AS (AS) [25] were maintained in RPMI1640/10%FCS. Confluent cultures were detached with trypsine or EDTA and split. ASML cells are NK resistant, AS cells are highly NK susceptible [34].

Antibodies are listed in Additional file 7.

Exosomes were separated by ultracentrifugation and sucrose density gradient [28]. In brief, cells were cultured (48 h) in serum-free medium. Cleared supernatants (2×10min, 500 g, 1×20min, 2000 g, 1x30min, 10000 g) were centrifuged (90 min, 100000 g) and washed (PBS, 90 min, 100000 g). Crude exosome preparations were suspended in 2.5 M sucrose, overlaid by a continuous sucrose gradient (0.25 M-2 M) and centrifuged (15 h, 150000 g). To exclude an impact of sucrose gradient centrifugation on exosome activity, sucrose gradient-purified exosomes were compared with the 100000 g pellet that was filtered through 0.20 μm membranes to remove, at least, larger non-exosome vesicles. Comparative analyses of sucrose-gradient enriched and 1000000 g/0.2 μm filtered exosomes are shown in Additional file 8. As we did not observe impaired activity of sucrose-gradient enriched exosomes, all other experiments were performed with the latter exosome population. Where indicated, exosomes were prepared after rhodamine-DHPE or SP-Dio_18_(3) (Invitrogen, Karlsruhe, Germany) labeling (60 min, 4°C). Relative fluorescence intensity was evaluated at 540 nm excitation, 590 nm emission or 497 nm excitation, 513 nm emission, respectively, and adjusted to rhodamine-DHPE or SP-Dio_18_(3) standards. ASML-exosomes have been characterized for protein composition [26], mRNA and miRNA content http://www.ncbi.nlm.nih.gov/geo/query/acc.cgi?acc=GSE34739.

### Cell and rat tissue preparation

Heparinized peripheral blood was collected by heart puncture. PBL were collected after Ficoll-Hypaque gradient centrifugation. PEC were collected flushing the peritoneal cavity with 10 ml PBS/heparin. BMC were collected from femora and tibiae, flushing the bones with 5 ml PBS. SC and LNC were obtained by pressing the organs through fine gauze. Where indicated, cells were CFSE (Invitrogen, Karlsruhe, Germany) labeled. Subpopulations were enriched by magnetic-bead sorting (Miltenyi, Mönchen-Gladbach, Germany). DC were generated *in vitro* from BMC. BMC (2×10^6^) were cultured in 10 cm diameter Petri dishes in 10 ml RPMI1640, supplemented with 10 ng/ml rrGM-CSF and 2 ng/ml rrIL-4. On day 3 of culture, additional 10 ml medium was added, exchanging half of the medium on day 6. Loosely adherent cells, harvested after 8d, were seeded in new Petri dishes in 10 ml medium containing 1 μg/ml LPS for 24 h to induce DC maturation. Matured DC were harvested on day 9, washed and loaded in serum-free RPMI with ASML-lysate (lysate of 3 cells/1 DC, overnight, 37°C).

### Exosome binding and uptake

Exosome binding/uptake *in vitro* was evaluated after 1 h-12 h co-incubation of dye-labeled exosomes with leukocytes. To differentiate between binding and uptake, bound exosomes were removed by two acid washes (PBS/HCl, pH 2.5) (stripping) evaluating exosome uptake by flow cytometry after fixation and permeabilization. When evaluating exosome binding structures on leukocytes or exosome targeting structures, leukocytes were incubated with the indicated antibodies for 30 min at 4°C and washed 2× with an excess of PBS. Dye-labeled exosomes, incubated with antibodies for 30 min at 4°C, were resuspended in 50 ml of PBS and centrifuged for 90 min at 100000 g. Antibody-coated washed cells / exosomes were co-incubated for 2 h at 4°C, washed 2-times with an excess of PBS and immediately analyzed by flow cytometry. The short incubation time at 4°C, though resulting in a lower binding rate, is mandatory to avoid exosome uptake.

Flow cytometry followed routine procedures. Where indicated, cells were fixed and permeabilized. Apoptosis was determined by AnnV/PI staining. Cell cycling was determined by CFSE dilution in labeled leukocytes. Samples were analyzed by a FACSCalibur and the Cell Quest Program.

### Immunofluorescence

Sections (7 μm) of shock frozen tissues from rats that had received an i.v. injection of 200 μg dye-labeled exosomes were counterstained with HE or marker-specific antibodies according to routine procedures. Digitized images were generated using a Carl Zeiss LSM780 confocal microscope and software Carl Zeiss Axioview Rel. 4.6.

### Proliferation assay

LNC and SC were titrated (2×10^5^-2.5×10^4^ cells/well) in 96 well plates with/without 10^4^ ASML lysate-loaded DC and/or 40 μg/ml exosomes. Cells were cultured for 3d adding 10 μCi/ml ^3^H-thymidine during the last 16 h. ^3^H-thymidine incorporation was evaluated in a β-counter.

### Cytotoxicity assays

CTL activity was evaluated after stimulating LNC with ASML lysate for 7d in RPMI/10%FCS/10U IL2/ml. ASML cells and, as control, syngeneic lymphoblasts (ConA-stimulated LNC) were used as targets. NK activity was evaluated after 2d of culture of SC or NKR-P1B^+^ cells [53] (magnetic bead separation) in the presence of 100U IL2/ml using AS cells as target. Cytotoxicity was evaluated using the JAM assay [54]. In brief, ^3^H-thymidine-labeled target cells (1×10^4^/well) were seeded on titrated numbers (1x10^6^-2.5×10^5^) of effector cells in 96 well plates. After 6 h at 37°C, plates were harvested, and radioactivity was determined in a β-counter. Cytotoxicity is presented as % cytotoxicity = 100 × (counts in control well - counts in test well) / (total counts/well).

### ELISA

IgM secretion as evaluated according to standard ELISA protocols. Plates were coated with 10 μg/ml anti-rIgM (overnight, 4°C). After washing and blocking, supernatants of cultures as described above were seeded on the plates overnight. Plates were washed (PBS/0,01% Tween20) and biotinylated anti-rIgM (2 μg/ml in PBS/0.5%BSA) was added. After incubation (2 h, 37°C), washing (6x, PBS/0.01%Tween20) and streptavidin-alkaline phosphatase enzyme-conjugate incubation (45 min, room temperature), BCIP/NBT substrate was added. OD (triplicates) was measured at 495 nm.

### Migration assay

Cells were seeded in the upper part of a Boyden chamber in 50 μl RPMI/0.1%BSA with/without 40 μg/ml exosomes. The lower part, separated by a 5 μm pore size polycarbonate-membrane (Neuroprobe, Gaithersburg, MD, USA) contained 30 μl RPMI/20%FCS. Leukocytes in the lower chamber were counted after 4 h. Where indicated, cells were pre-incubated with antibody (10 μg/ml). Migration is presented as % of input cells.

### *In vivo* experiments

BDX rats received an i.v. injection of SP-Dio_18_(3)-labeled exosomes (200 μg/rat). After 24 h, rats were bled, sacrificed and lymphatic organs were excised. For vaccination, rats received 3 subcutaneous injections of 2×10^6^ ASML-lysate-loaded DC in 10d intervals. Starting with the first DC application, rats received 500 μg ASML-exosomes, 2-times/week. Rats were sacrificed 3d after the 3^rd^ DC application. Animal experiments were Government-approved (Baden-Württemberg, Germany).

### Statistical analysis

Assays were repeated at least 3 times. P-values <0.05 (two-tailed Student’s t-test) were considered significant.

## Abbreviations

AS: BSp73AS; ASML: BSp73ASML; BMC: Bone marrow cells; CTL: Cytotoxic T cells; DC: Dendritic cells; HSP: Heat shock proteins; MDSC: Myeloid-derived suppressor cells; Mϕ: Macrophages; LNC: Lymph node cells; NK: NK cells; PEC: Peritoneal exudate cells; SC: Spleen cells; TC: Thymocytes; TIL: Tumor infiltrating leukocytes; Th: Helper T cells; T_reg_: Regulatory T cells.

## Competing interests

Authors declare no competing interest.

## Authors' contribution

DZ and SR performed and evaluated experiments, MWB helped with manuscript editing, MZ planned, performed and evaluated experiments and wrote the manuscript. All authors read and approved the final manuscript.

## Supplementary Material

Additional File 1 Examples of ASML-exosome binding and uptake. Click here for file

Additional File 2 Impact of ASML-exosomes on major leukocyte subset marker expression. Click here for file

Additional File 3 Tumor-exosomes and expression of signal transduction molecules in spleen cells. Click here for file

Additional File 4 Tumor-exosomes and B cell activation. Click here for file

Additional File 5 Tumor-exosomes and adhesion molecule expression in lymph node cells. Click here for file

Additional File 6 **Tumor-exosomes and lymphocyte activation *in vivo*.
** Click here for file

Additional File 7 Antibodies. Click here for file

Additional File 8 Comparison of 100000 g pellet and sucrose density gradient-enriched exosomes. Click here for file
