# Supplementary material for: Tumor-exosomes and leukocyte activation: an ambivalent crosstalk
Source: Cell Commun Signal. 2012 Nov 28;10:37. doi: 10.1186/1478-811X-10-37 (PMC3519567; doi:10.1186/1478-811X-10-37)
Supplement: Additional File 1 — Examples of ASML-exosome binding and uptake. [file 1478-811X-10-37-S1.pdf]

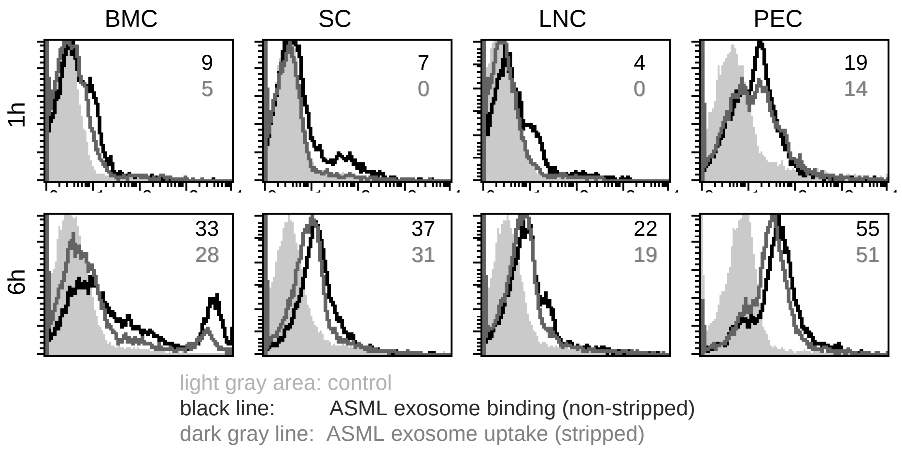

Add.File 1 <u>Tumor-exosome uptake by leukocytes</u> BMC, SC, LNC and PEC were incubated at 37oC with RhDHPE-labeled ASML-exosomes for 1h or 6h. Exosome binding and uptake (fluorescence after 2 acid washes) was evaluated by flow-cytometry. Representative examples are shown. The percentage of cells with bound or uptaken exosomes are indicated.
