# Supplementary material for: Tumor-exosomes and leukocyte activation: an ambivalent crosstalk
Source: Cell Commun Signal. 2012 Nov 28;10:37. doi: 10.1186/1478-811X-10-37 (PMC3519567; doi:10.1186/1478-811X-10-37)
Supplement: Additional File 2 — Impact of ASML-exosomes on major leukocyte subset marker expression. [file 1478-811X-10-37-S2.pdf]

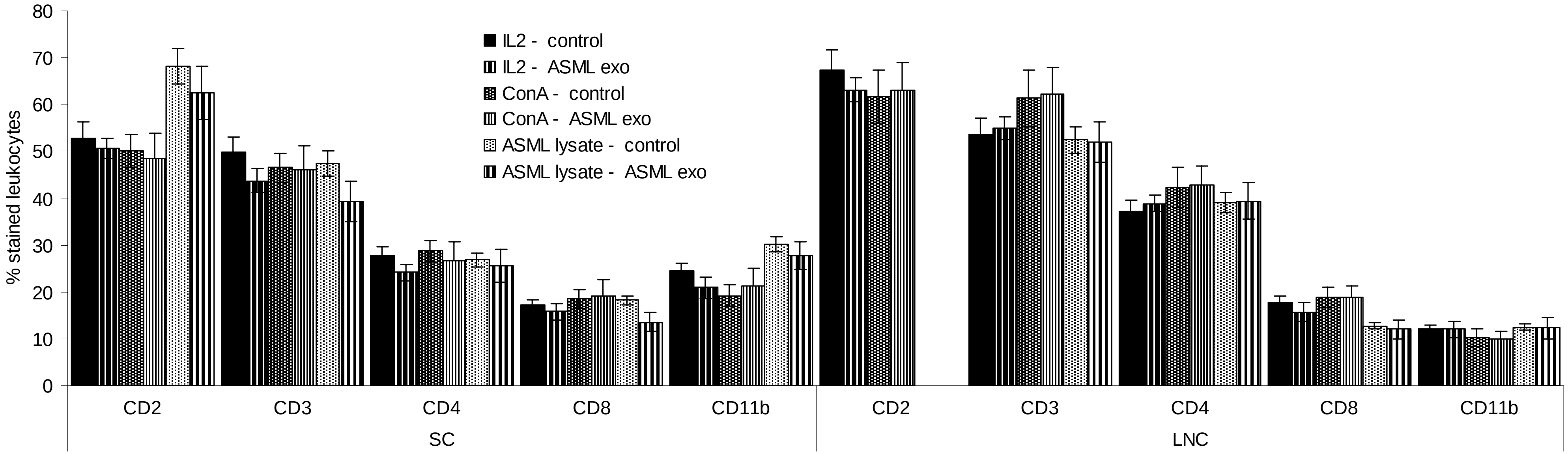

Add.File 2 <u>Impact of ASML-exosomes on major leukocyte subsets</u> SC and LNC were stimulated with IL2, ConA or ASML-lysate with/without ASML-exosomes. Leukocyte marker expression was evaluated by flow-cytometry: Mean percent±SD (3 experiments) of stained cells; No significant differences in the presence of ASML-exosomes.
