# Supplementary material for: Tumor-exosomes and leukocyte activation: an ambivalent crosstalk
Source: Cell Commun Signal. 2012 Nov 28;10:37. doi: 10.1186/1478-811X-10-37 (PMC3519567; doi:10.1186/1478-811X-10-37)
Supplement: Additional File 3 — Tumor-exosomes and expression of signal transduction molecules in spleen cells. [file 1478-811X-10-37-S3.pdf]

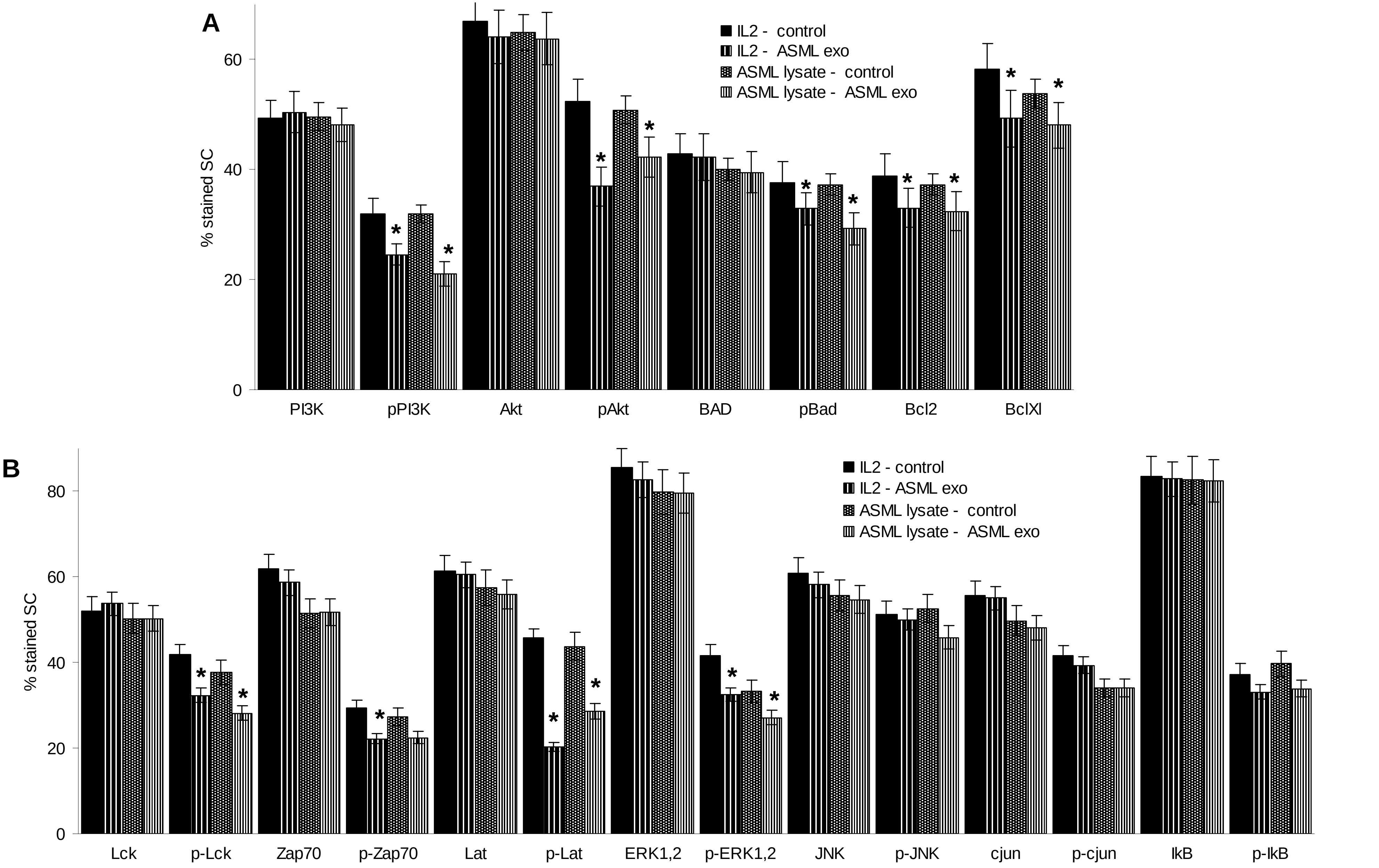

Add.File 3 Tumor exosomes and expression of signal transduction molecules in spleen cells SC were cultured for 48h in the presence of IL2 or ASML-lysate with/without ASML-exosomes. Expression of molecules involved in (A) anti-apoptotic signaling and (B) TCR activation was evaluated by flow-cytometry: Mean percent±SD (3 experiments) of stained cells; Significant differences in the presence of ASML-exosomes: *.
