# Supplementary material for: Tumor-exosomes and leukocyte activation: an ambivalent crosstalk
Source: Cell Commun Signal. 2012 Nov 28;10:37. doi: 10.1186/1478-811X-10-37 (PMC3519567; doi:10.1186/1478-811X-10-37)
Supplement: Additional File 4 — Tumor-exosomes and B cell activation. [file 1478-811X-10-37-S4.pdf]

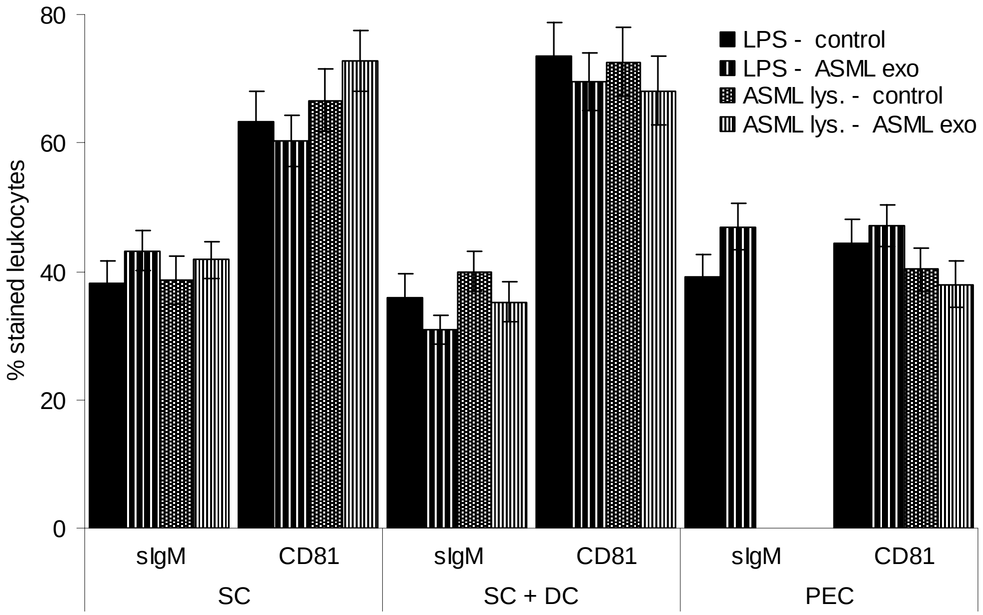

Add.File 4 Tumor exosomes and B cell activation SC and PEC were stimulated for 48h by LPS or ASML-lysate with/without ASML-exosomes. Where indicated, cultures contained ASML-lysate-pulsed DC. Expression of sIgM and CD81 was evaluated by flow-cytometry: Mean percent±SD (3 experiments) of stained cells.
