# Supplementary material for: Tumor-exosomes and leukocyte activation: an ambivalent crosstalk
Source: Cell Commun Signal. 2012 Nov 28;10:37. doi: 10.1186/1478-811X-10-37 (PMC3519567; doi:10.1186/1478-811X-10-37)
Supplement: Additional File 5 — Tumor-exosomes and adhesion molecule expression in lymph node cells. [file 1478-811X-10-37-S5.pdf]

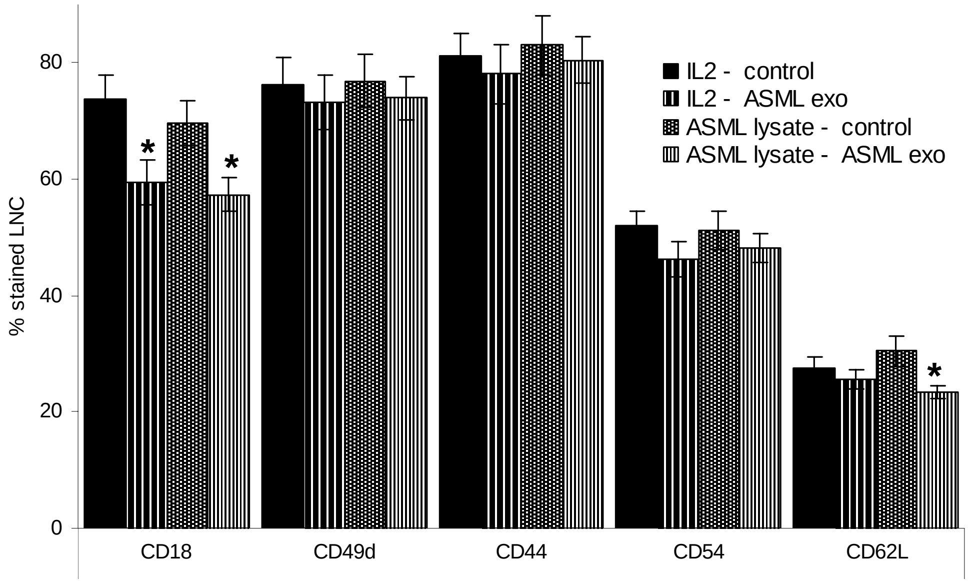

Add.File 5 Tumor-exosomes and adhesion molecule expression in lymph node cells LNC were stimulated for 24h with IL2 or ASML-lysate with/without ASML-exosomes. Expression of adhesion molecules was evaluated by flow-cytometry: Mean percent±SD (3 experiments) of stained cells; Significant differences in cultures containing ASML-exosomes: *.
