# Supplementary material for: Tumor-exosomes and leukocyte activation: an ambivalent crosstalk
Source: Cell Commun Signal. 2012 Nov 28;10:37. doi: 10.1186/1478-811X-10-37 (PMC3519567; doi:10.1186/1478-811X-10-37)
Supplement: Additional File 6 — Tumor-exosomes and lymphocyte activation in vivo. [file 1478-811X-10-37-S6.pdf]

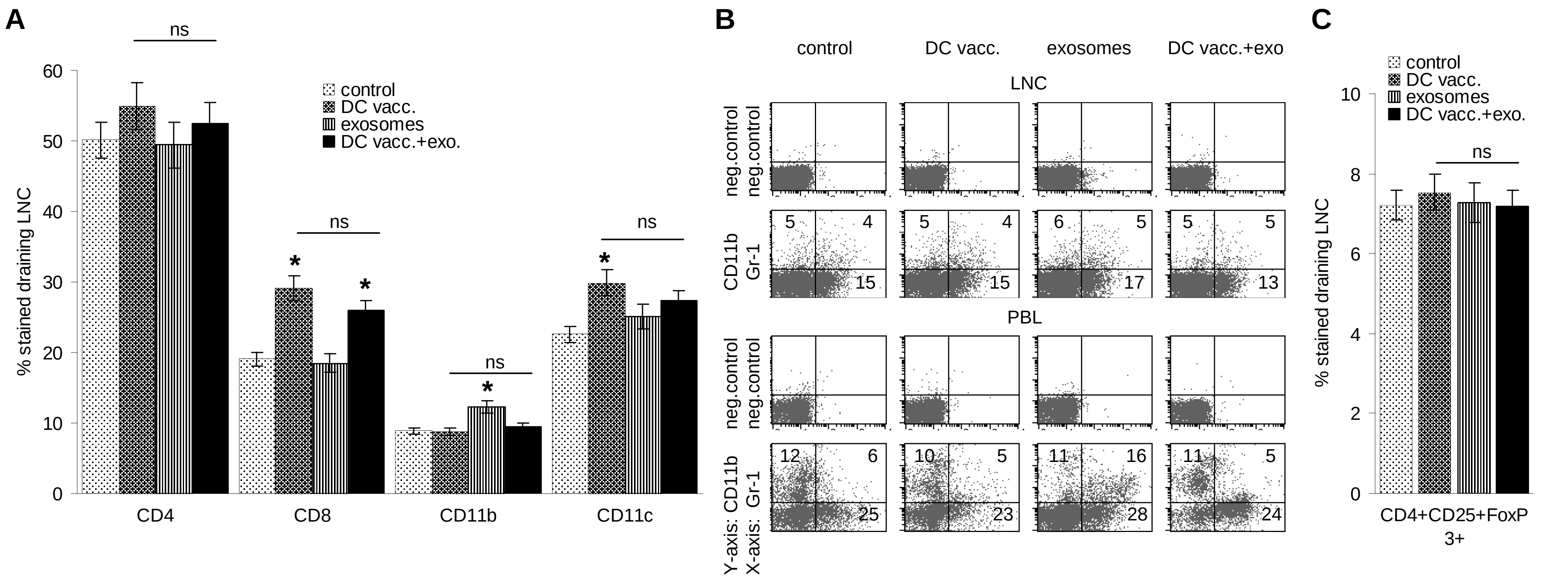

Add.File 6 <u>*In vivo* impact of ASML-exosomes on leukocyte activation</u> Rats received 3-times 2x106 DC, subcutaneously and/or 7-times 500µg ASML-exosomes, i.v. as described in MM. Rats were sacrificed 3d after the 3rd DC application. (A) Leukocyte subset distribution (flow cytometry, mean±SD, 3 rats), (B) MDSC (representative example), (C) Treg (flow cytometry, mean±SD, 3 rats). (A,C) Significant differences to lymphocytes from untreated rats: *, differences between lymphocytes from rats receiving DC or DC plus ASML-exosomes are indicated as ns (not significant) or s (significant, p <0.01).
