# Supplementary material for: Tumor-exosomes and leukocyte activation: an ambivalent crosstalk
Source: Cell Commun Signal. 2012 Nov 28;10:37. doi: 10.1186/1478-811X-10-37 (PMC3519567; doi:10.1186/1478-811X-10-37)
Supplement: Additional File 7 — Antibodies. [file 1478-811X-10-37-S7.pdf]

Aditional File 7
Antibodies

Antibody	Supplier	Antibody	Supplier
Akt	BD[1], Heidelberg, G[2]	ezrin	SantaCruz, Heidelberg,G
Bad	SantaCruz, Heidelberg,G	fyn	SantaCruz, Heidelberg,G
Bak	BD, Heidelberg,G	Gr1	BD, Heidelberg, G
Bax	BD, Heidelberg, G	granzymeB	BD, Heidelberg, G
Bcl2	BD, Heidelberg, G	IgM (rat)	Dianova, Hamburg,G
BclXl	BD, Heidelberg, G	IFNγ	BD, Heidelberg, G
Bid	BD, Heidelberg, G	IκB	Cell Signaling, Frankfurt, G
Casp3,activ.	BD, Heidelberg, G	IL2	BD, Heidelberg, G
Casp8	BD, Heidelberg, G	JNK	BD, Heidelberg, G
Casp9,cleaved	SantaCruz, Heidelberg,G	LAT	SantaCruz, Heidelberg,G
CD2	clone Ox34 (EACC)[3]	lck	SantaCruz, Heidelberg,G
CD3	clone R73 (ref 1)	MHCII	clone Ox6 (EACC)
CD4	clone Ox35 (EACC)	NKR-P1B	BD, Heidelberg, G
CD8	clone Ox8 (EACC)	p-Akt	BD, Heidelberg, G
CD9	clone B2C11, DSHB[4] BD, Heidelberg, G	p-Bad	SantaCruz, Heidelberg,G
CD11a	BD, Heidelberg, G	p-c-jun	Santa Cruz, Heidelberg, G.
CD11b	clone Ox42 (EACC)	p-ERK1/2	BD, Heidelberg, G
CD11c	clone Ox41 (EACC)	p-ezrin	SantaCruz, Heidelberg,G
CD18	BD, Heidelberg, G	p-FAK	BD, Heidelberg, G
CD25	clone Ox38 (EACC)	p-fyn	Santa Cruz, Heidelberg, G
CD28	clone Ox61 (EACC)	PI3K	Santa Cruz, Heidelberg, G
CD44	clone Ox50 (EACC)	p-PI3K	Santa Cruz, Heidelberg, G
CD44v6	clone A2.6 (ref.2)	p-IκB	Cell Signaling, Frankfurt, G
CD49c	clone Ralph3.1, DSHB, BD, Heidelberg, G	p-JNK	BD, Heidelberg, G
CD49d	BD, Heidelberg, G	p-LAT	BD, Heidelberg, G
CD53	clone Ox44 (EACC)	PLCγ	BD, Heidelberg, G
CD54	BD, Heidelberg, G	p-PLCγ	Cell Signaling, Frankfurt, G
CD61	BD, Heidelberg, G	p-lck	Santa Cruz, Heidelberg, G
CD62L	BD, Heidelberg, G	p-src	Santa Cruz, Heidelberg, G
CD63	BD, Heidelberg, G	p-Stat5	Santa Cruz, Heidelberg, G
CD80	BD, Heidelberg, G	p-syk	Santa Cruz, Heidelberg, G
CD81	BD, Heidelberg, G	p-ZAP70	Santa Cruz, Heidelberg, G
CD86	BD, Heidelberg, G	SDF1	Santa Cruz, Heidelberg, G
CD95	BD, Heidelberg, G	SHP1	Cell Signaling, Frankfurt, G
CD106	BD, Heidelberg, G	SHP2	Cell Signaling, Frankfurt, G
CD151	(ref.3)[5]	Smac/Diablo	BD, Heidelberg, G
CD178	BD, Heidelberg, G	src	Santa Cruz, Heidelberg, G
c-jun	SantaCruz, Heidelberg,G	TNFα	BD, Heidelberg, G
CXCR4	SantaCruz, Heidelberg,G	TRAF4	BD, Heidelberg, G
CyclinD3	Santa Cruz, Heidelberg, G	TRAIL	BD, Heidelberg, G
cytoch.C	BD, Heidelberg, G	Tspan8	clone D6.1 (ref.2)
ERK1/2	BD, Heidelberg, G	XIAP	BD, Heidelberg, G
FAK	BD, Heidelberg, G	ZAP70	BD, Heidelberg, G
FoxP3	Biozol, Eching, G		
mIgG, mIgM, rabbitIgG, goatIgG, hamster IgG, Streptavidin (unlabeled, HRP-, AP-, FITC-, Cy3-, PE-, APC-labeled)			Dianova, Hamburg, G

[1] BD: Becton Dickinson, [2] G: Germany, [3] EACC: European Animal Cell Culture Collection, Porton Down, UK, [4] DSHB: Developmental studies hybridoma bank, University of Iowa, Iowa, USA,[5] Besides the rabbit anti-CD151 antibody (ref.3), a corresponding antibody was generated in guinea pig.

References

1. Lawetzky A, Tiefenthaler G, Kubo R, Hünig T. **Identification and characterization of rat T cell subpopulations expressing T cell receptors alpha/beta and gamma/delta.** *Eur J Immunol* 1990, **20**:343-349.
2. Matzku S, Wenzel A, Liu S, Zöller M. **Antigenic differences between metastatic and non-metastatic rat tumor variants characterized by monoclonal antibodies.** *Cancer Res* 1989, **49**:1294-1299.
3. Claas C, Wahl J, Orlicky D, Karaduman H, Schnölzer M, Kempf T, Zöller M. **The tetraspanin D6.1A and its molecular partners on rat carcinoma cells.** *Biochem J* 2005, **389**:99-110.
