# Supplementary material for: Tumor-exosomes and leukocyte activation: an ambivalent crosstalk
Source: Cell Commun Signal. 2012 Nov 28;10:37. doi: 10.1186/1478-811X-10-37 (PMC3519567; doi:10.1186/1478-811X-10-37)
Supplement: Additional File 8 — Comparison of 100000 g pellet and sucrose density gradient-enriched exosomes. [file 1478-811X-10-37-S8.pdf]

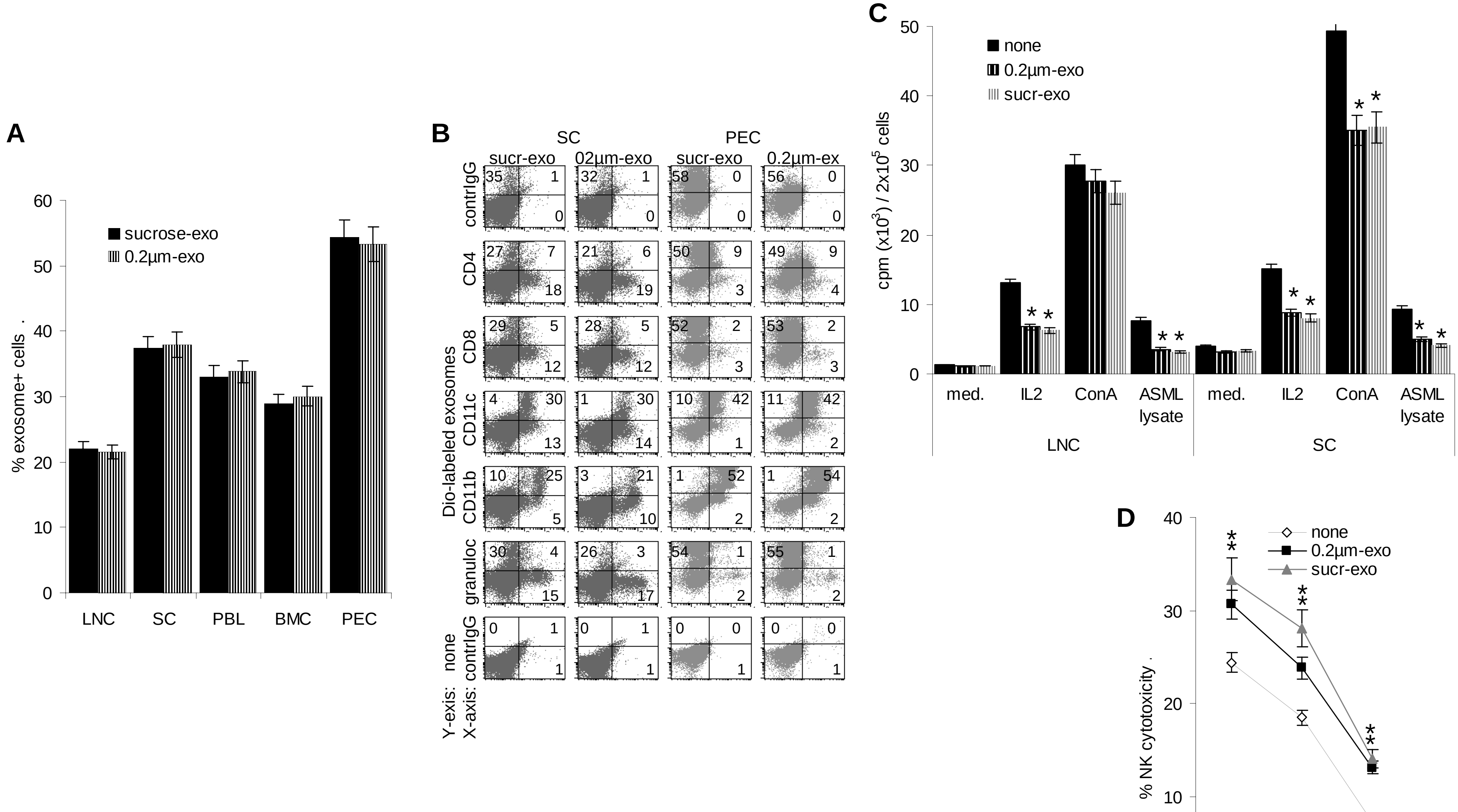

Add.File 8 Comparison of 100000g- and sucrose gradient-enriched exosomes ASML-exosomes in the 100000g pellet were filtered through 0.2µm membranes (0.2µm-exo) and compared with sucrose-gradient enriched exosomes (sucr-exo). (A,B) SP-Dio18(3)-labeled 0.2µm-exo and sucr-exo were co-cultured with LNC, SC, BMC, PBL and PEC for 6h. Where indicated, SC and PEC were stained for leukocyte markers. Binding was evaluated by flow cytometry. The mean percent±SD of exosome+ cells (2 experiments) and representative examples are shown. (C) LNC and SC were stimulated for 72h with IL2, ConA or ASML-lysate in the presence of 0.2µm- or sucro-exosomes. Mean±SD (triplicates) of 3H-thymidine incorporation is shown. (D) SC were cultured for 48h in the presence of 100U IL2/ml in the presence of 0.2µm- or sucro-exosomes. NK cytotoxicity was evaluated with 3H-thymidine labeled AS target cells. (C,D) Significant differences in cultures containing exosomes are indicated: *. (A,C,D) The comparison between 0.2µm- and sucro-exosomes did not reveal significant difference.
